# The Structure of Liquid and Glassy Carbamazepine

**DOI:** 10.3390/qubs6040031

**Published:** 2022-11-15

**Authors:** Chris J. Benmore, Angela Edwards, Oliver L. G. Alderman, Brian R. Cherry, Pamela Smith, Daniel Smith, Stephen Byrn, Richard Weber, Jeffery L. Yarger

**Affiliations:** 1 Advanced Photon Source, Argonne National Laboratory, Argonne, IL 60439, USA; 2 School of Molecular Sciences, Arizona State University, Tempe, AZ 85281, USA; 3 Rutherford Appleton Laboratory, Harwell Campus, Didcot OX11 0QX, UK; 4 Improved Pharma, West Lafayette, IN 47906, USA; 5 Materials Development, Inc., Arlington Heights, IL 60004, USA

**Keywords:** carbamazepine, amorphous, liquid structure, glass structure, X-ray diffraction, hydrogen bonding

## Abstract

To enhance the solubility of orally administered pharmaceuticals, liquid capsules or amorphous tablets are often preferred over crystalline drug products. However, little is known regarding the variation in bonding mechanisms between pharmaceutical molecules in their different disordered forms. In this study, liquid and melt-quenched glassy carbamazepine have been studied using high energy X-ray diffraction and modeled using Empirical Potential Structure Refinement. The results show significant structural differences between the liquid and glassy states. The liquid shows a wide range of structures; from isolated molecules, to aromatic ring correlations and NH-O hydrogen bonding. Upon quenching from the liquid to the glass the number of hydrogen bonds per molecule increases by ~50% at the expense of a ~30% decrease in the close contact (non-bonded) carbon-carbon interactions between aromatic rings. During the cooling process, there is an increase in both singly and doubly hydrogen-bonded adjacent molecules. Although hydrogen-bonded dimers found in the crystalline states persist in the glassy state, the absence of a crystalline lattice also allows small, hydrogen-bonded NH-O trimers and tetramers to form. This proposed model for the structure of glassy carbamazepine is consistent with the results from vibrational spectroscopy and nuclear magnetic resonance.

## 1. Introduction

Amorphous pharmaceuticals often possess greater solubility and bioavailability than their crystalline forms [1,2]. However, competition between different intermolecular bonding arrangements in liquid and amorphous pharmaceuticals, associated with the manufacturing, storage temperature and exposure to humidity, can have a substantial effect on the structure phase stability [3,4]. From a crystalline standpoint carbamazepine (CBZ) has served as a model compound for groups studying of crystal polymorphism [5]. Dissolution rates of the crystalline forms I, III and the dihydrate, correlate strongly with solubility. This leads to significant differences in the bioavailability between the anhydrous and dihydrate forms after oral administration. In the fall of 1998 ~70 million tablets containing carbamazepine were withdrawn from the market because of a reported clinical failure, based on the fact that the dihydrate was formed instead of form III [3].

Here we have characterized crystalline and glassy forms of CBZ using Differential Scanning Calorimetry (DSC), Fourier Transform Infrared Spectroscopy (FTIR), and solid-state Nuclear Magnetic Resonance (ssNMR). The structure of the liquid and glassy states have also been studied using high energy X-ray diffraction (HEXRD) and modeling using Empirical Potential Structure Refinement (EPSR). Previous total scattering experiments on CBZ have compared the amorphous pair distribution functions (PDFs) of CBZ with known crystalline forms, and good agreement has been found with spherical nanocrystalline particles of CBZ form III crystallites of 4.5 nm in size [6]. However, the authors acknowledged that a truly a homogeneous amorphous structure with short-range molecular CBZ III-like packing ‘could not be ruled out’. Here, we argue that the assumption of single (or multiple) crystalline forms in itself, can be problematic when modeling amorphous structures. This has notably been the case in interpreting the PDF for water [7], where three crystalline forms were manipulated to reproduce the liquid PDF but the model contained several thermodynamic and scattering flaws [8,9]. Indeed, Wright has previously detailed an analogous debate on “the great crystallite vs random network controversy” for understanding the structure of inorganic amorphous and glassy solids [10]. While [10] found that early crystallite theory is untenable, it is acknowledged the likelihood of finding frozen in local crystallite motifs above the fictive temperature, and that the original random network theory of glasses by Zachariasen is only a first-order approximation. The number, size distribution, and volume fraction of crystalline-like motifs will always be a major question.

The accuracy and nature of the modeling procedure will always be an important aspect of understanding the structure of amorphous materials. For organic amorphous molecules, the situation is even more complex as variations in molecular shape are common-place when a long-range lattice is not present as an additional constraint. Moreover, since amorphous forms are by definition metastable a variety of structures can be formed, depending on fictive temperature or preparation history. This has recently been demonstrated in the X-ray PDF of amorphous Indomethacin, which is particularly sensitive to the preferred orientations of the chlorobenzyl ring [11]. Depending on the fabrication conditions (namely humidity) in some cases the chlorobenzyl ring is found to have no preferred torsional angle in the amorphous form, while in others there is evidence of distinct isomer orientations found in the crystal forms. This is derived from competition between subtle intra- and inter-molecular bonding configurations and is most clearly reflected in the intensity of the first sharp diffraction peak of the X-ray structure factor S(Q). This latter observation underscores the importance of comparing the measured total scattering data and model in reciprocal space as well as real space. Here we show the Monte Carlo simulation approach used by EPSR is well suited to modeling small rigid molecules with a specific geometry and a limited number of bonding sites such as CBZ.

## 2. Materials and Methods

Experiments on both the liquid and glassy forms of CBZ were subject to the samples quickly degrading over time. Partial crystallization of the high-temperature melt was observed to occur within 5–10 min using most techniques, especially in sealed containers where the absorbed water could not escape. In addition, the conversion of the glass to the crystalline dihydrate form occurred on a similar time-scale in humid environments at room the temperature, as water was absorbed from the atmosphere.

### 2.1. Nuclear Magnetic Resonance Spectroscopy

Solution state NMR of the as-received carbamazepine (Alfa-Aesar 98% purity) was dissolved in CDCl_3_ and collected on a Bruker 500 MHz NEO spectrometer with a 5 mm iProbe. The solid-state NMR data were collected on a Varian VNMRS (18.8 T) operating at a Larmor frequency of 799.84 MHz and 201.14 MHz for ^1^H and ^13^C respectively. A 1.6 mm Varian T3 high speed MAS probe was employed with a MAS speed of 35 kHz for the crystalline material. To eliminate crystallization concerns, the amorphous material (produced by the same method as used in high energy X-ray diffraction experiments) was only spun to 20 kHz MAS, to limit frictional heating. Sample temperature thereby stayed well below Tg = 46 °C. Cross polarization (CP) with a ^13^C B_1_ field of 62.5 kHz and a 10% linear ramp with a 1.5 ms contact time on the ^1^H CP contact pulse matching to the ^−1^ condition, depending on MAS speed. ^1^H decoupling at a B_1_ field of 117.6 kHz with time proportional phase modulation (TPPM) was used during acquisition. Data were collected with a sweep width of 100 kHz, an acquisition time of 20 msec, and a recycle delay of 720 s or 15 s for the crystalline and amorphous material, respectively. Peak positions were fit using Top-Spin 4.0 for CDCl3 solution, CBZ III and dihydrate. CBZ glass peaks were fit using Gaussian deconvolution in OriginPro, see Supplementary Materials.

### 2.2. Vibrational Spectroscopy

Fourier transform infrared spectroscopy (FTIR) was performed using a Bruker Alpha II FTIR Instrument equipped with a platinum single reflection attenuated total reflection (ATR) module with a monolithic diamond interface, temperature stabilized deuterated triglycine sulfate wideband near-infrared detector, and an integrated certified reference standard. Spectra of form III crystalline CBZ stock, (Alfa Aesar ~98%, and Sigma Aldrich > 99.9%), as well as the thermally annealed form I of both, CBZ dihydrate (CBZ·2H_2_O) recrystallized from form III stock (Alfa Aesar, 98%) in supersaturated EtOH formed by heating to 80 °C then the addition of 20% (*v/v*) H_2_O antisolvent during cooling to 4 °C and melt quenched amorphous products from each form) were collected in ambient conditions (~25 °C, 1 atm, in air) from 400 to 4000 cm^−1^ in increments of 2 cm^−1^ for a total of 128 scans, with a background collected between each sample using the same specifications. Spectra were minimally processed with a zero-baseline correction applied at lower frequencies by interpolation, and normalization to equalize intensities for comparison.

### 2.3. Thermal Analysis

Differential scanning calorimetry measurements were performed on a TA Instruments Discovery 2500 DSC with a constant dry nitrogen flow > 200 mL/min. The baseline, forward, and reversing heat capacity were calibrated using sapphire disc standards, while the cell constant was calibrated using In wire standard (Strem chemicals, 99.9985%). 2–3 mg samples (form III crystalline CBZ stock, Alfa Aesar ~98%, and Sigma Aldrich > 99.9%, as well as the thermally annealed form I and melt quenched amorphous products from each form, and the CBZ·2H_2_O recrystallized as described above) were loaded in non-hermetic Al crucibles. The use of a non-hermetic pan (critically, under N_2_ flow in the DSC cell) is necessary to prevent increased pressure at higher temperatures, which has been demonstrated to increase the degradation of CBZ to iminostilbene (IMB) [12]. Sample expansion in these pans at higher temperatures risks leaking CBZ through the lid, so the maximum temperature used for CBZ samples in standard pans was 215 °C (~24 °C above the melting point of form I). Cyclic DSC experiments were carried out at a heating scan rate of 10 °C/min, with variable cooling rates between 1–10 °C/min.

### 2.4. High-Energy X-ray Diffraction

The X-ray pair distribution function (PDF) method is an established technique for the cintermediate-range both local and intermediate range ordering of disordered organic materials, providing details of molecular structure at the atomic level. Powdered carbamazepine samples (Aldrich > 98% purity) were loaded into 1.5 mm diameter, unsealed thin walled (0.1 mm) capillaries, and heated to 230 °C, 40 °C above the melting point for the X-ray measurements lasting 2 min. The glass was immediately quenched from the melt into liquid nitrogen and measured at room temperature. The high-energy X-ray measurements performed were on beamline 6-ID-D at the Advanced Photon Source at Argonne National Laboratory. The setup and correction procedures have been previously described in detail [13]. Experiments were carried out using a monochromatic X-ray beam E = 100 keV (λ = 0.124 Å) collimated to a square 0.5 mm cross section, and the scattered beam was measured using a Varex (CT4343) area detector. NIST CeO_2_ powder was used for sample-detector distance calibration, which was set to 360 mm in order to balance resolution and Q-range. An additional time-resolved humidity experiment on amorphous cryoground, fluffy carbamazepine powder was performed at 80% relative humidity (RH, Electro-Tech Systems, Inc. Glenside, PA, USA, model 5503) using a Pilatus 2M CdTe detector after drying CBZ at 20% RH for 2 h.

The total X-ray scattering data were analyzed as described previously using Fit2D and PDFgetX2 [14,15]. In brief, geometrical, polarization, background and attenuation corrections were applied to all data sets. For the Varex detector dark current corrections were applied. The total X-ray structure factor S(Q) and differential pair distribution function D(r) have been defined in reference [16]. Additional masking was needed for the Pilatus 2M detector due to the dead zones between detector elements and residual trapped excited states.

### 2.5. Empirical Potential Structure Refinement

**Figure 1.**
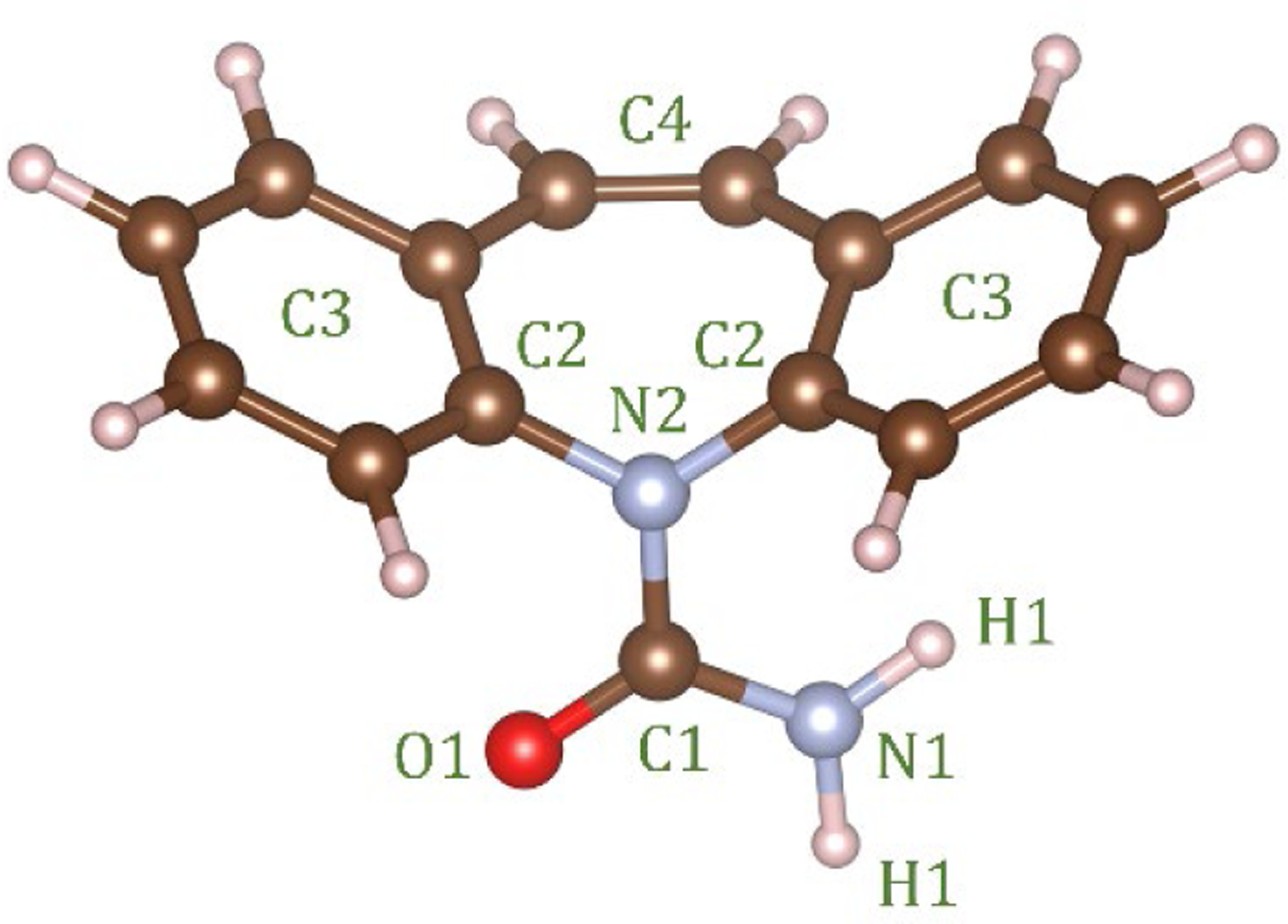
Structure of the carbamazepine molecule with the atom labels used in the EPSR simulation. In EPSR C3 corresponds to the 5 carbon atoms in the ring.

Empirical Potential Structural Refinement (EPSR) modeling [17,18] was used to obtain atomistic models of liquid and glassy carbamazepine (C_15_H_12_N_2_O, see Figure 1), based on the high-energy X-ray diffraction data. The EPSR simulations were performed on 100 molecules within a cubic box under periodic boundary conditions, using atomic number densities of 0.1025 atomsÅ^−3^ for the glass and 0.1000 atomsÅ^−3^ for the liquid. The parameters for the Lennard-Jones reference potentials are given in Table 1. The starting configuration was a random array of molecules and following initial Monte Carlo equilibration, the empirical potential term was refined to improve agreement with scattering data, Once the goodness-of-fit parameter was minimized between the model and the experimental S(Q), structural data were collected over ensembles of at least 5000 configurations. Enabling rotations along the N2-C1 and C1-N1 bonds resulted in no significant improvement in the fits. While the EPSR fit to the data does not necessarily give a unique structural 3D configuration of molecules, it does provide an important insight into the types of interactions that are likely in the disordered state.

## 3. Results

### 3.1. Sample Characterization

**Figure 2.**
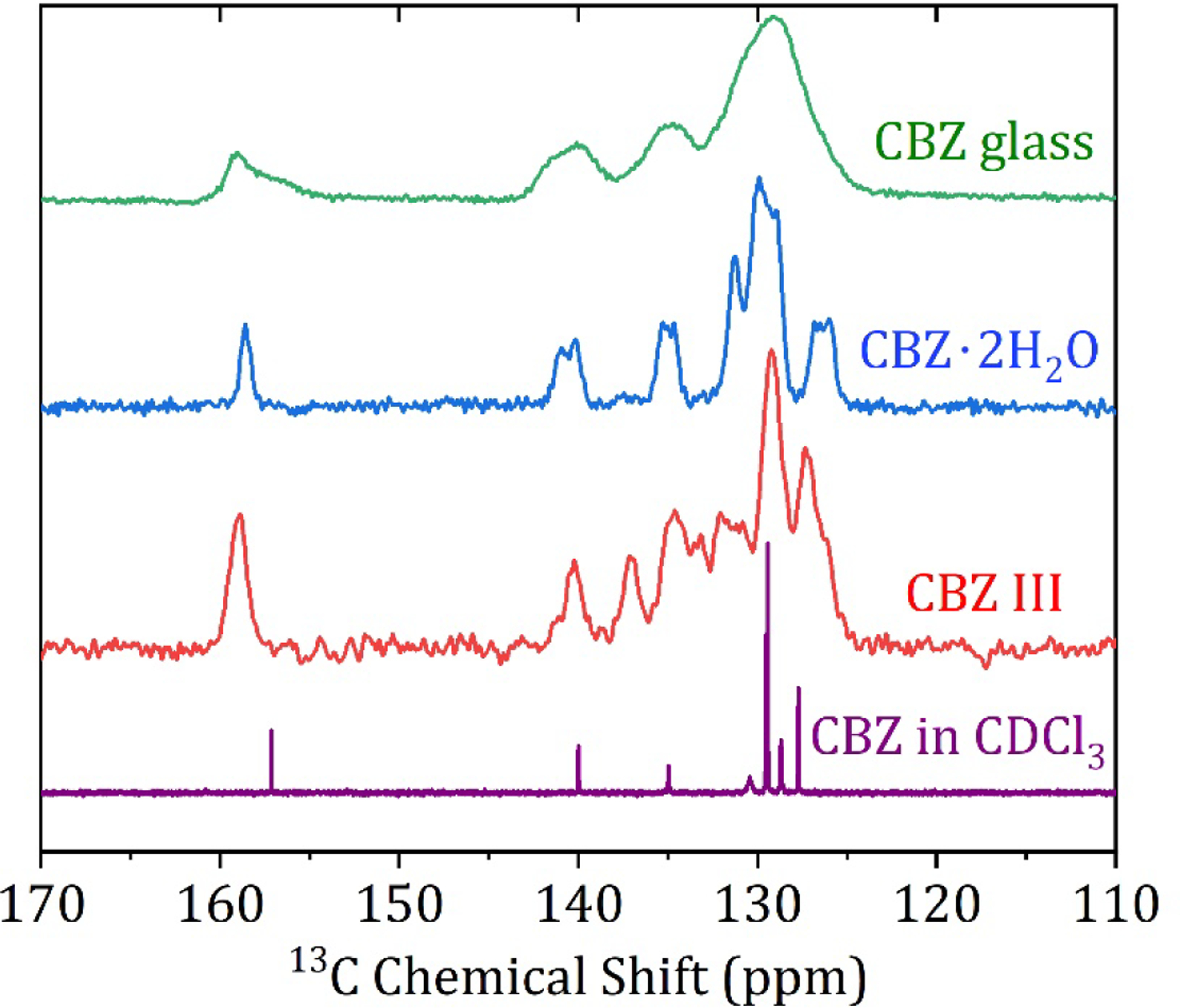
Solid State ^13^C CP-MAS NMR spectra of amorphous carbamazepine, crystalline CBZ dihydrate, crystalline CBZ III, and solution spectrum in CDCl_3_.

In contrast to the melt-quenched glass measured on the X-ray beamline, to perform ssNMR on amorphous CBZ, the freshly melt-quenched sample had to be ground into a powder and loaded in ambient conditions. The remaining powder was stored at 4 °C for the time it took to load the rotor into the probe and optimize d1 to set up the ^1^H-^13^C CP-MAS experiment (Figure 2, top). Though the measurement was made at only 20 kHz spinning speed to minimize frictional heating, FTIR and DSC measurements were promptly performed to verify the absence of the thermal degradation of CBZ samples and absence of nucleation or recrystallization in amorphous CBZ samples.

**Figure 3.**
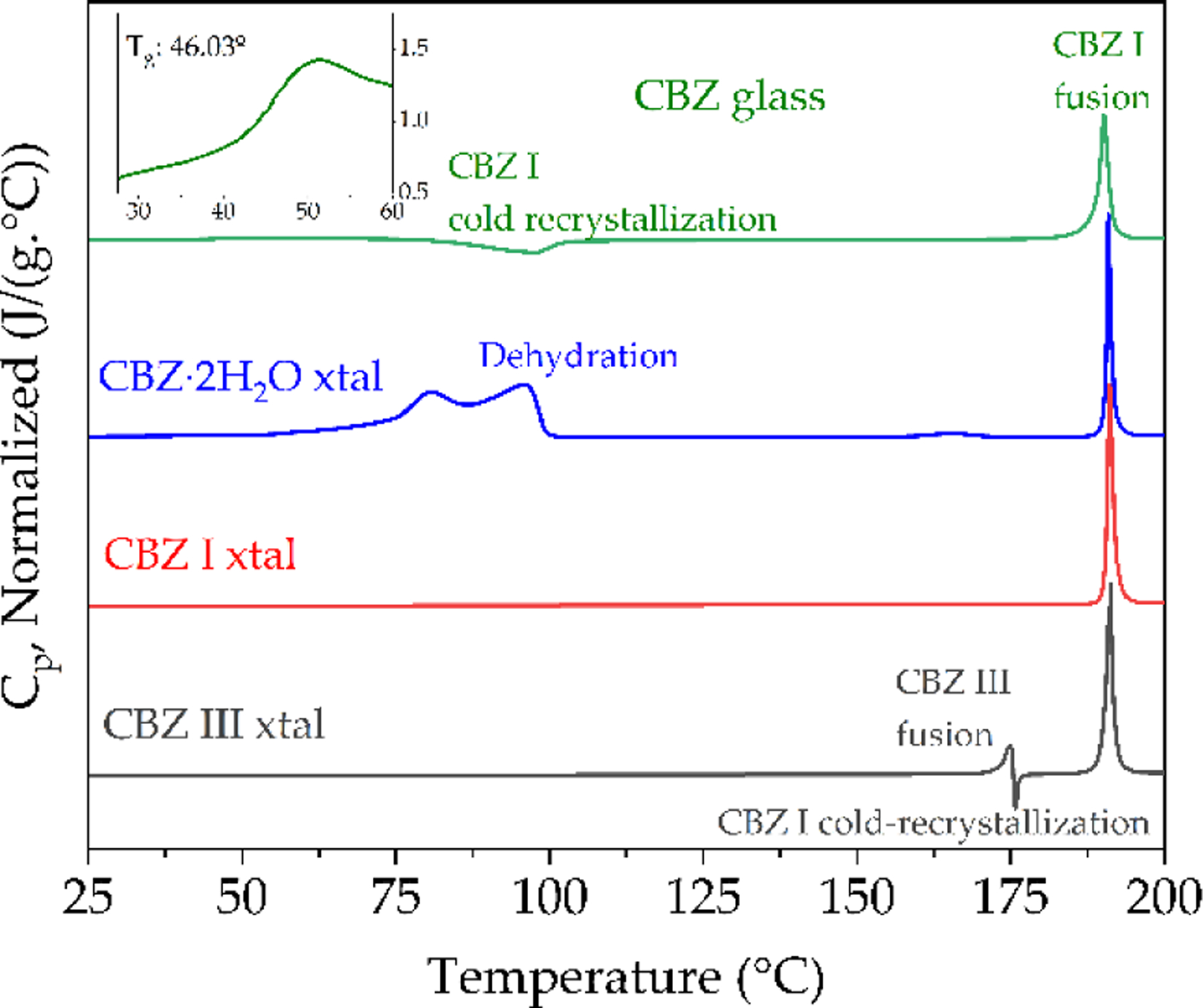
DSC thermograms of the first heating cycle at 10 °C/min under dry N_2_ of amorphous CBZ with inset enlarging the range from 30–60 °C to show glass transition; crystalline CBZ dihydrate, CBZ I (triclinic) and CBZ III (p-monoclinic).

**Figure 4.**
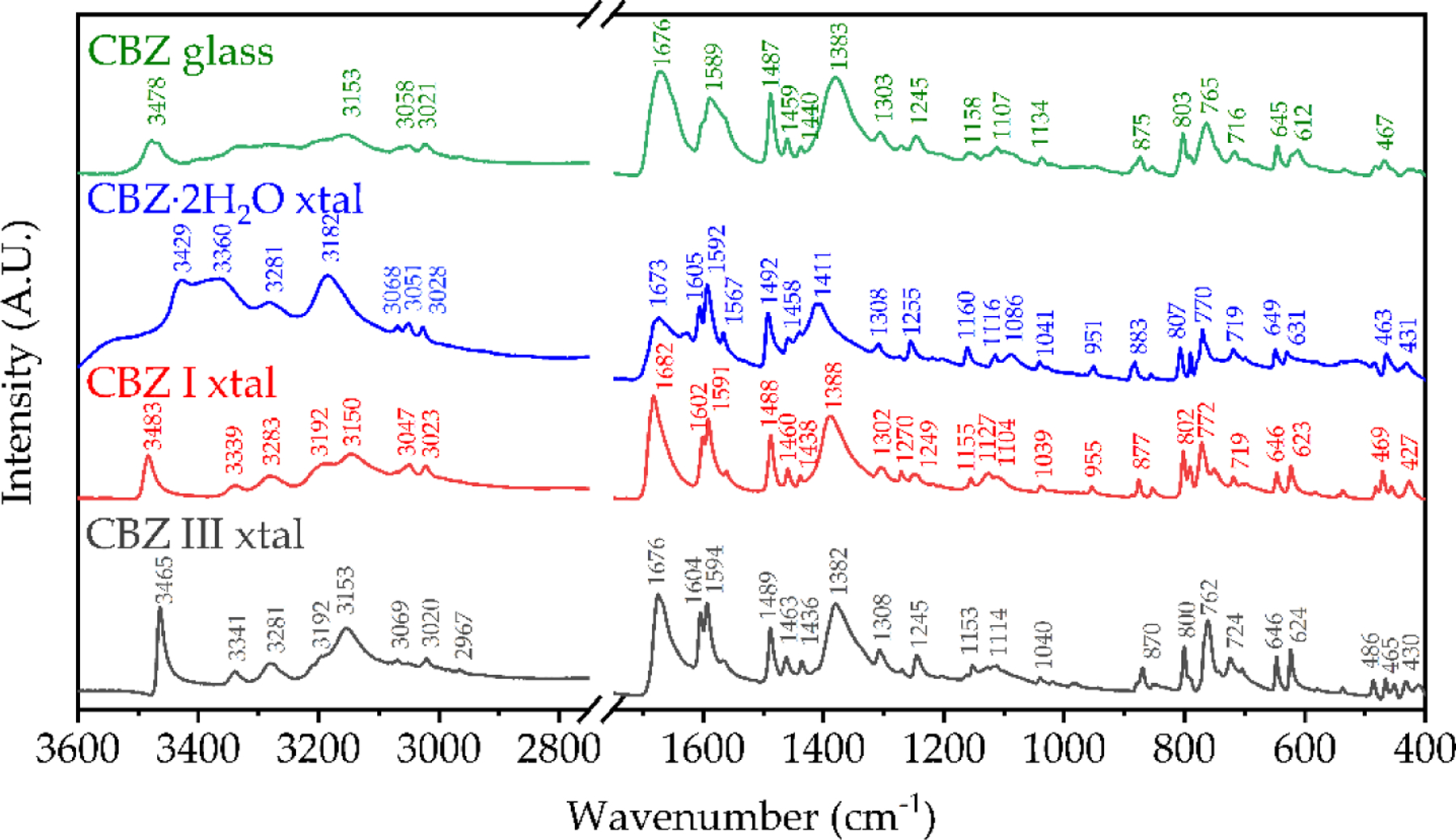
FTIR-ATR spectra of amorphous CBZ, and crystalline CBZ dihydrate, form I (triclinic) and form III (p-monoclinic) with main peaks labeled.

The solution ^13^C chemical shifts of CBZ in CDCl_3_ and the ^1^H-^13^C CP-MAS ^13^C chemical shifts of the crystalline form III and dihydrate agree well with published values assigned using HETCOR and COSY experiments (Figure 2 middle and lower curves) [5], summarized in Table 2. The amorphous CBZ spectrum (Figure 2, top) has very broad peaks, such that the majority of the fused ring structure peaks are not resolved. ^1^H-^13^C CP-MAS is not precisely quantitative on a natural abundance sample due to the differential efficiency of magnetization transfer rates with different heteronuclear dipolar interaction strengths within the sample, so deconvolution to correctly integrate percentages of states quantitatively is not possible [19,20]. Nonetheless, some qualitative conclusions are apparent from the ^1^H-^13^C CP-MAS spectrum of glassy carbamazepine, and a deconvoluted spectrum is given in the supplementary materials. Importantly, the carboxamide peak at 159.3 ppm has a clear shoulder at 157.6 ppm. In solution, the carboxamide peak chemical shift is 157.2 ppm, while the crystalline polymorphs range from 159.0–159.9 ppm for polymorphs I-IV, and 158.5 ppm for the dihydrate. There is also an asymmetry to the peak at 140.1 ppm, where a shoulder is visible at 141.9 ppm. The origin of these spectral details is discussed in comparison to the X-ray pdf data and modeling in the next section. It is clear from the ssNMR data in Figure 2 when taken with the DSC and FTIR data (Figure 3 and Figure 4, respectively) that neither the crystalline polymorphs nor the amorphous CBZ are converted in detectible quantities to the dihydrate at ambient conditions (~22 °C, 20–30% R.H.) during the experimental time frame used for these experiments.

Immediately after initiating ssNMR experiments the amorphous CBZ was subsequently quickly loaded in duplicate in DSC pans (Figure 3, top) and during the run of the first sample the FTIR-ATR measurement was taken (Figure 4). The importance of quickly measuring amorphous CBZ when handled in ambient conditions has been extensively characterized [21,22], to prevent nucleation and recrystallization as well as decarboxamination to the IMB degradation product that happens more readily in air [23]. Dołęga et al. [22] have used temperature-controlled FTIR in tandem with DSC and HPLC to study the kinetics of non-isothermal cold-crystallization of the quench-cooled Carbamazepine, as well as quantify the amount of IMB in amorphous CBZ samples and re-crystallized CBZ polymorphs, demonstrating that even small IMB percentages can be detected by either method. The fast heating rate before melt-quenching increases the degradation temperature, which is observed to have an onset by conventional TGA heating at 10 °C/min of ~240 °C (data not shown) in agreement with literature values [20]. Critically, the absence of the endotherm at 140 °C attributed to the fusion of the eutectic mixture of IMB and CBZ [23] indicates that the bulk CBZ as received and the amorphous CBZ samples in this study did not undergo decomposition during preparation, handling, or measurements (Figure 3, transitions summarized in Table 2).

The FTIR-ATR spectra of CBZ I and III agree with the literature values of Grzesiak et al. [24], though their hermetically sealed crucible DSC results show some variation with higher melting transitions for cold-recrystallized form I from form III melting that was not observed in this study (Figure 3, bottom and Table 3) and other recent studies [19]. The FTIR spectra of CBZ contaminated with IMB critically showed a loss of the asymmetric amide stretch vibration at 3478 cm^−1^, with the formation of peaks at 3416 cm^−1^ and ~3190 cm^−1^ not present in the samples in this study (Figure 4, top).

### 3.2. High-Energy X-ray Diffraction and EPSR Modeling Results

**Figure 5.**
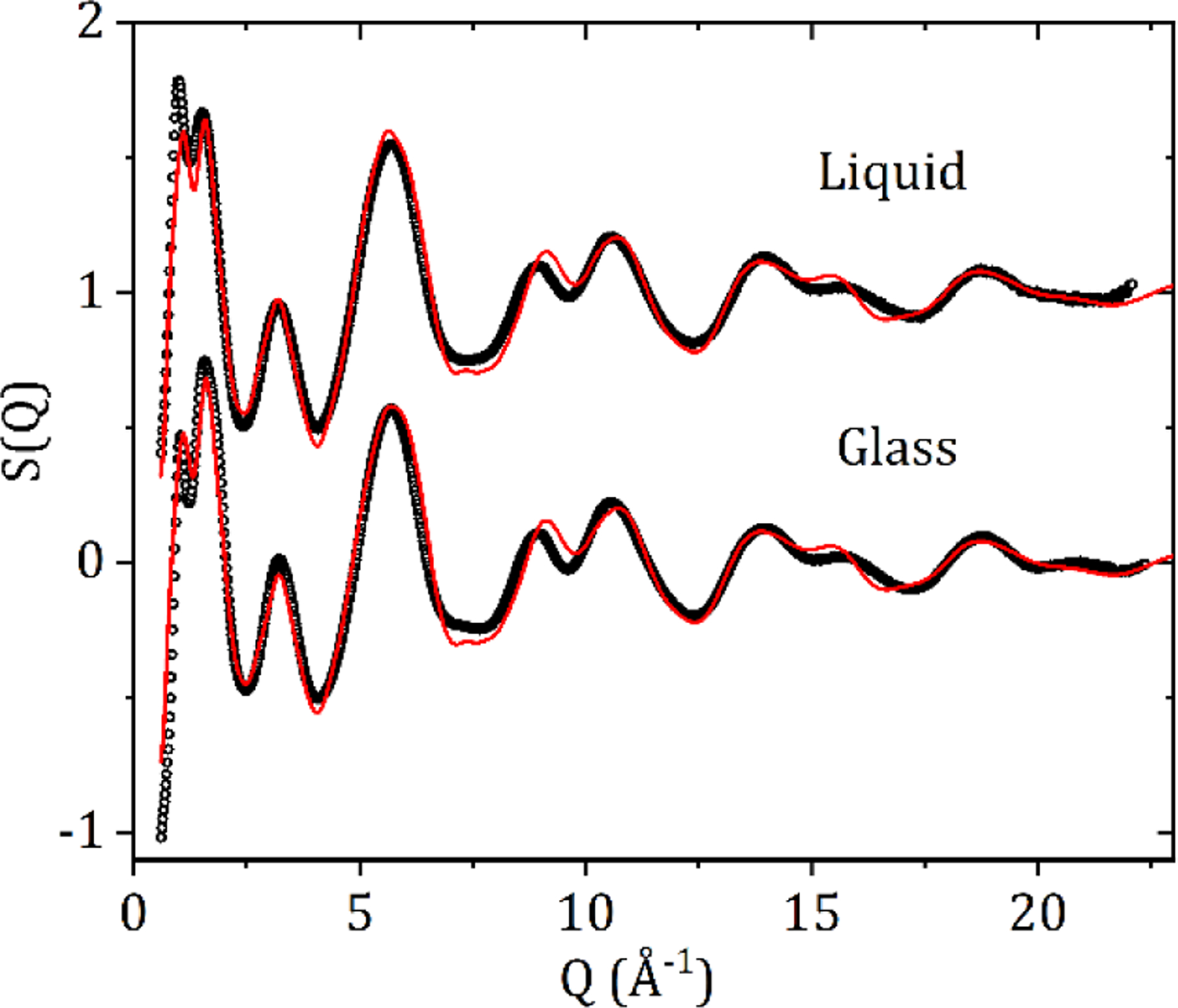
The X-ray total structure factors S(Q) and EPSR fits for liquid and glassy CBZ.

The measured X-ray total structure factors for liquid and glassy carbamazepine and the EPSR model fit are shown in Figure 5. The high-Q region is dominated by the intra-molecular scattering and the first double peak feature is strongly correlated with the inter-molecular packing arrangements and changes in density. Best fits were obtained for the glass using an atomic number density 2.5% higher than that of the liquid.

**Figure 6.**
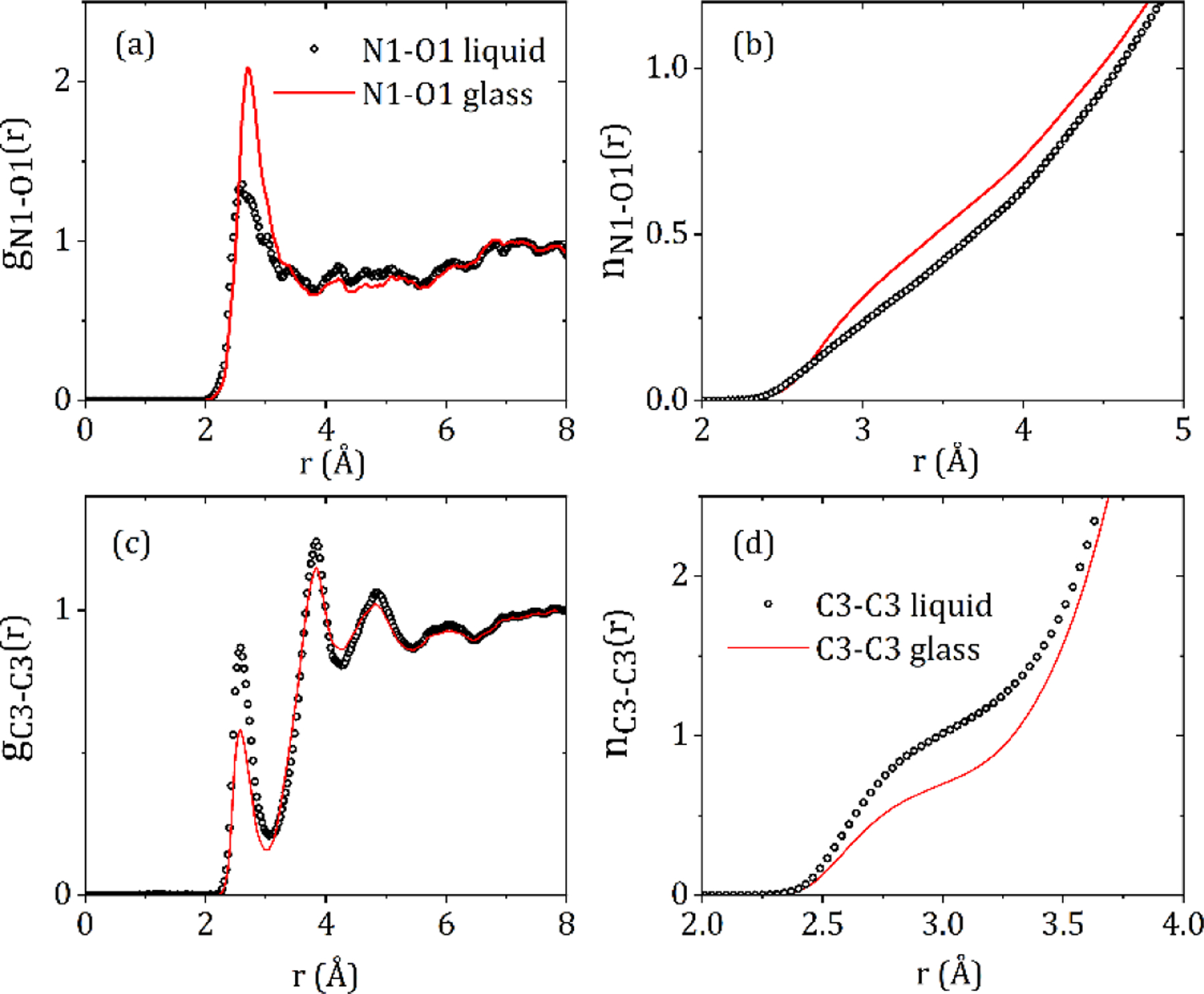
The (**a**) N1-O1 and (**c**) C3-C3 partial pair distribution functions, and the (**b**) N1-O1 and (**d**) C3-C3 running coordination numbers for liquid and glassy CBZ.

Since X-rays are scattered by electrons, the S(Q)’s and corresponding PDF’s are most sensitive to the heavier atoms and in particular the orientations of the carbon rings on the ‘wings’ of the molecules, labeled C3 in Figure 1. The intermolecular C3-C3 partial structure factor and corresponding C3-C3 partial pair distribution function showsa significant change between the liquid and glassy forms (see Figure 6). A distinct first inter-molecular peak at 2.58 Å and a second peak at 3.84 Å are both more intense in the liquid state, and indicate an increased number of close contacts between aromatic rings. The minima after first peak in g_C3-C3_(r) is at r = 3.06 Å, and corresponds to an average coordination number of n_C3-C3_ = 1.06 ± 0.03 in the liquid and 0.74 ± 0.02 in the glass. Given the distinct geometry of the carbamazepine molecule these molecular orientations in the model define the average NH-O hydrogen bond distances present. Hydrogen bonding is most clearly observed in the N1-O1 partial pair distribution function, which exhibits a well-defined peak at 2.9 Å. The minima after the first peak in g_N1-O1_(r) is at r = 4.0 Å, corresponds to an average coordination number of n~0.73 ± 0.02 in glass and 0.63 ± 0.02 in the liquid.

## 4. Discussion

**Figure 7.**
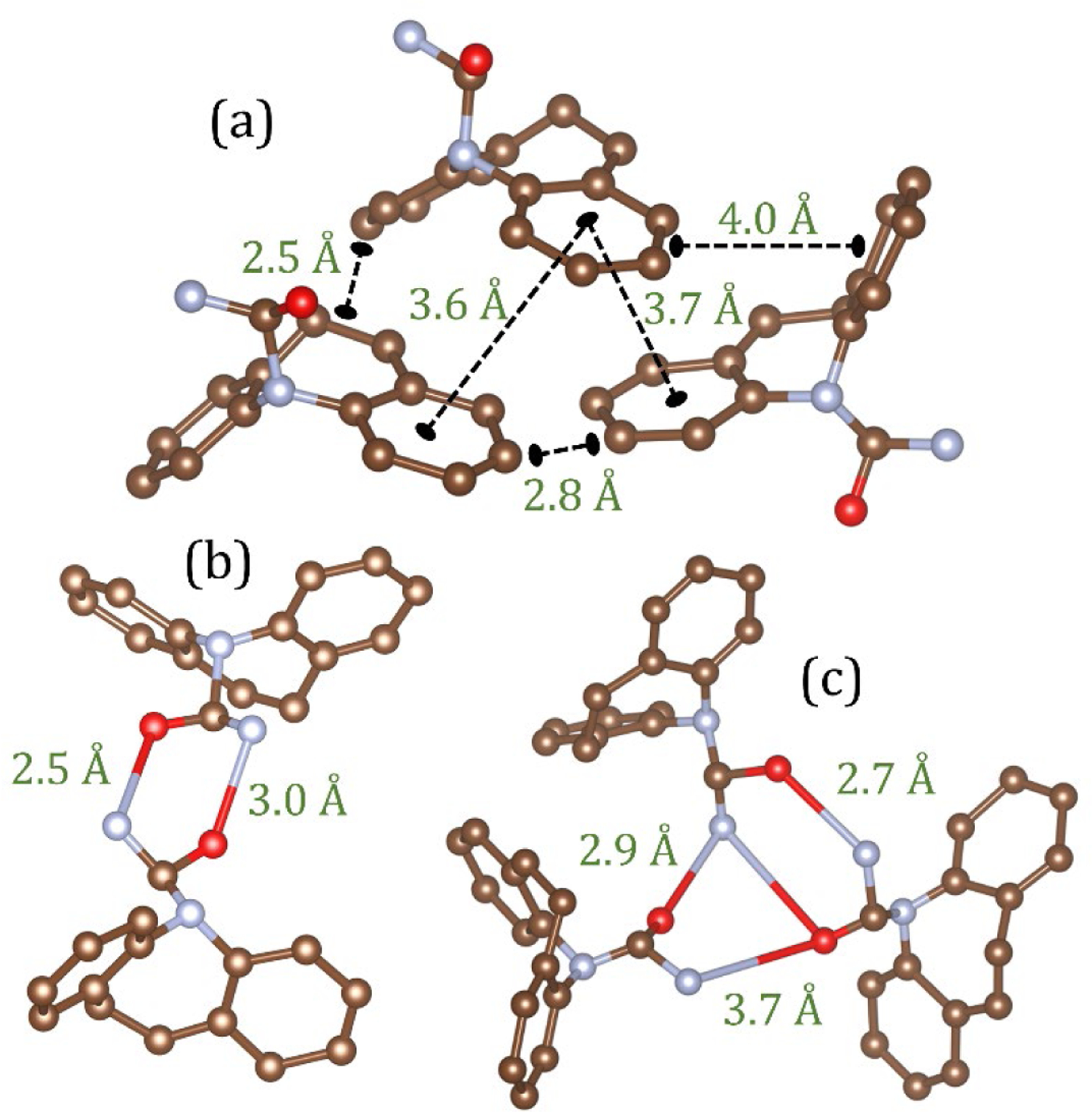
Snapshots of molecular configurations taken from the EPSR models and their associated distances. (**a**) An example of close contact C3-C3 interactions as a consequence of π-π interactions between aromatic rings in the liquid state model (**b**) A hydrogen bonded carbamazepine dimer and (**c**) trimer with shared NH-O bonds in the glass model.

In CBZ the four anhydrous crystals all contain similar molecular conformations with stabilities that are within 0.7 kcal/mol of each other [24]. In addition, all polymorphs have the same strong hydrogen bonding patterns containing dimers of carbamazepine molecules linked via pairs of NH-O hydrogen bonds. In crystalline carbamazepine, forms I and II have similar packing of dimers, with an offset π-π stacking of the aromatic rings as the main interaction between neighboring dimers. In forms III and IV aromatic rings form both π-π stacking and edge-to-face contacts in an interlocked packing arrangement. The stability order of these polymorphs at room temperature has been ranked as III > I > IV > II [24]. A previous study on amorphous carbamazepine has promoted a nanocrystalline model of a 3–4 molecular aromatic stack of hydrogen bonded dimers similar to that found in form III [6]. In the EPSR liquid model the first inter-molecular g_C3-C3_(r) peak at 2.58 Å can be attributed to non-bonded ring interactions, while the second peak at 3.84 Å is a distance more commonly associated with π-π stacking and edge-to-face contacts, as shown in Figure 7a.

**Figure 8.**
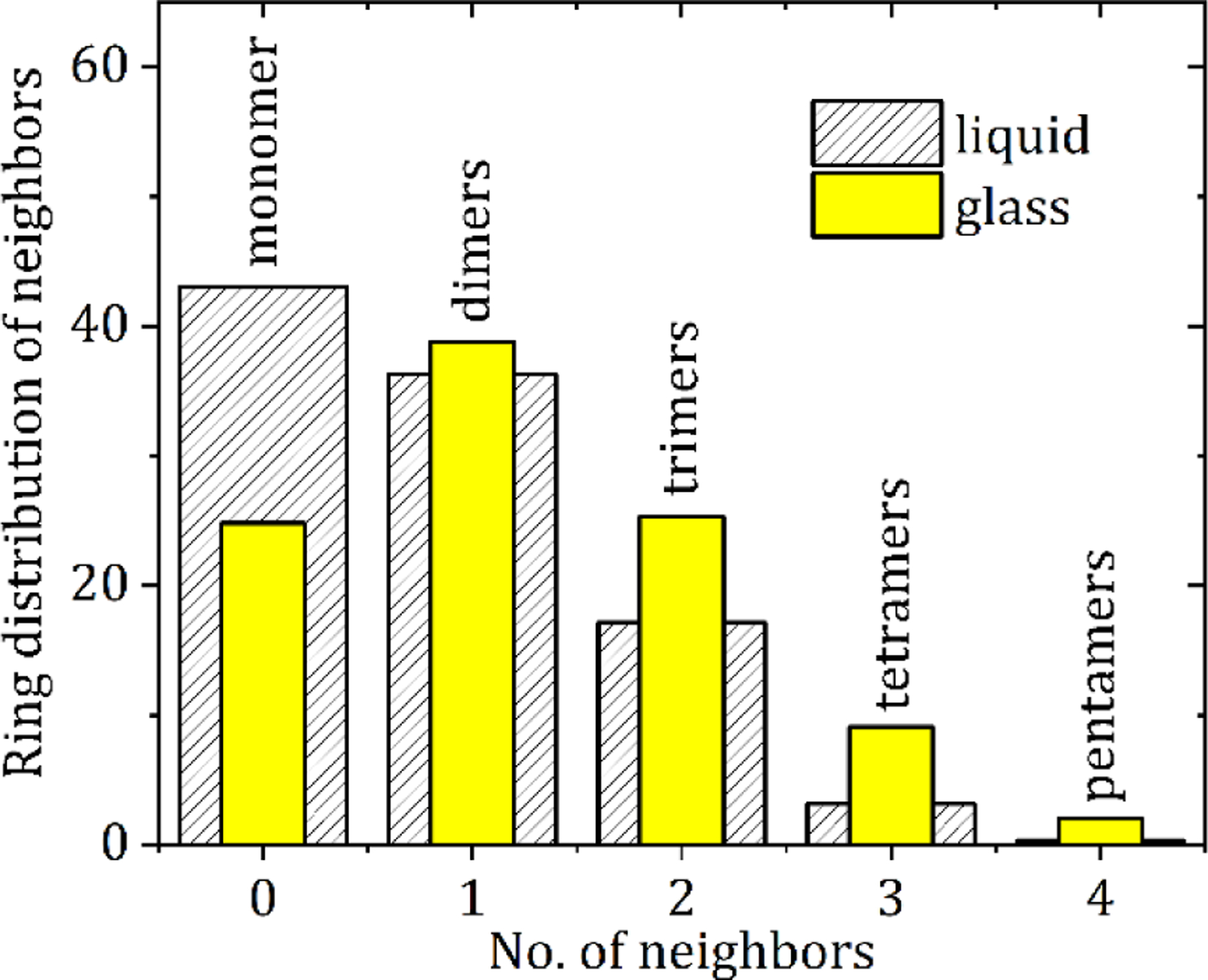
X-ray sensitive atom-atom distances between N and O atoms (depicted as blue and red respectively in Figure 7) up to 4.0 Å for liquid and glassy CBZ.

The average ring and chain distributions for NH-O hydrogen bonding in the EPSR model are shown in Figure 8, using an N-O cut off distance of 4.0 Å. Here the ring distributions show that this average is a mixture of isolated molecules and small clusters of 1 to 4 nearest neighbors. In the crystalline state there are only dimers are connected via two NH-O bonds and these configurations also persist in the liquid and glassy states (see Figure 7b). However, our model shows the liquid contains more single (non-hydrogen bonded) molecules, whereas the glass comprises mostly of dimers and small hydrogen bonded clusters. This is reflected in the average bonds per molecule, which increases from 0.8 ± 0.2 in the liquid model to 1.2 ± 0.2 in the glassy model. Given that there is only a 16% increase in the first peak of g_N1-O1_(r) between the liquid and glass, this reflects an increase in both singly and doubly hydrogen bonded adjacent molecules upon cooling. In addition to dimers, trimers, tetramers and pentamers also form via a shared hydrogen bonding arrangement. This is illustrated by the trimer in Figure 7c where one molecule shares OH-N bonds with two different molecules, a structural motif not found in the crystalline state.

The increase in hydrogen bonding upon cooling is also reflected in the ^1^H-^13^C CP-MAS ssNMR data (Figure 2), in which there is clear asymmetry in the peak at 140.1 ppm, with a shoulder at 141.9 ppm. In solution, there is only one peak at 139.9 ppm, whereas the solid-state chemical shift anisotropy separates these two carbons by 2–3 ppm depending on the polymorph due to the restriction of rotation about the N2-C1 bond in Figure 1 [5]. While there is no precise evidence of a trimeric (nor higher oligomers) structure from the ssNMR data, the shoulder peak at 157.6 ppm for the carboxamide ^13^C chemical shift suggests that there are indeed interactions that are not seen in any of the known solid states in which the dimeric interaction has a chemical shift of 159.0–159.9 ppm for anhydrous CBZ polymorphs. Moreover, this shoulder peak is clearly very broad, and while quantitative deconvolution is not possible for this natural abundance sample due to the experimental conditions, it is clear that the expected peak at 159.1 ppm corresponding to a mixture of dimeric structures is the same order of magnitude as the shoulder i.e., that the sum of the other states have a greater concentration to the expected dimeric structures (see supplementary materials for more information). There is also a clear distribution of states of the primary interaction via the carboxamide moiety observed in the FTIR spectrum of CBZ glass in Figure 4. The CBZ I and III polymorphs display sharp peaks for the NH_2_ asymmetric stretch at 3483 cm^−1^ and 3465 cm^−1^, respectively [25]. This vibrational mode is changed significantly for the dihydrate due to hydrogen bonding with water, giving two broad, overlapping peaks at 3429 cm^−1^ and 3360 cm^−1^. The glass has none of these features, with a broad single peak at 3478 cm^−1^, which is intermediate to the dimeric states of the two crystalline polymorphs but not blue-shifted as observed for the dihydrate outside of the bounds of the two most different triclinic (CBZ I) and p-monoclinic (CBZ III) forms used in this study.

### Hydration of CBZ

**Figure 9.**
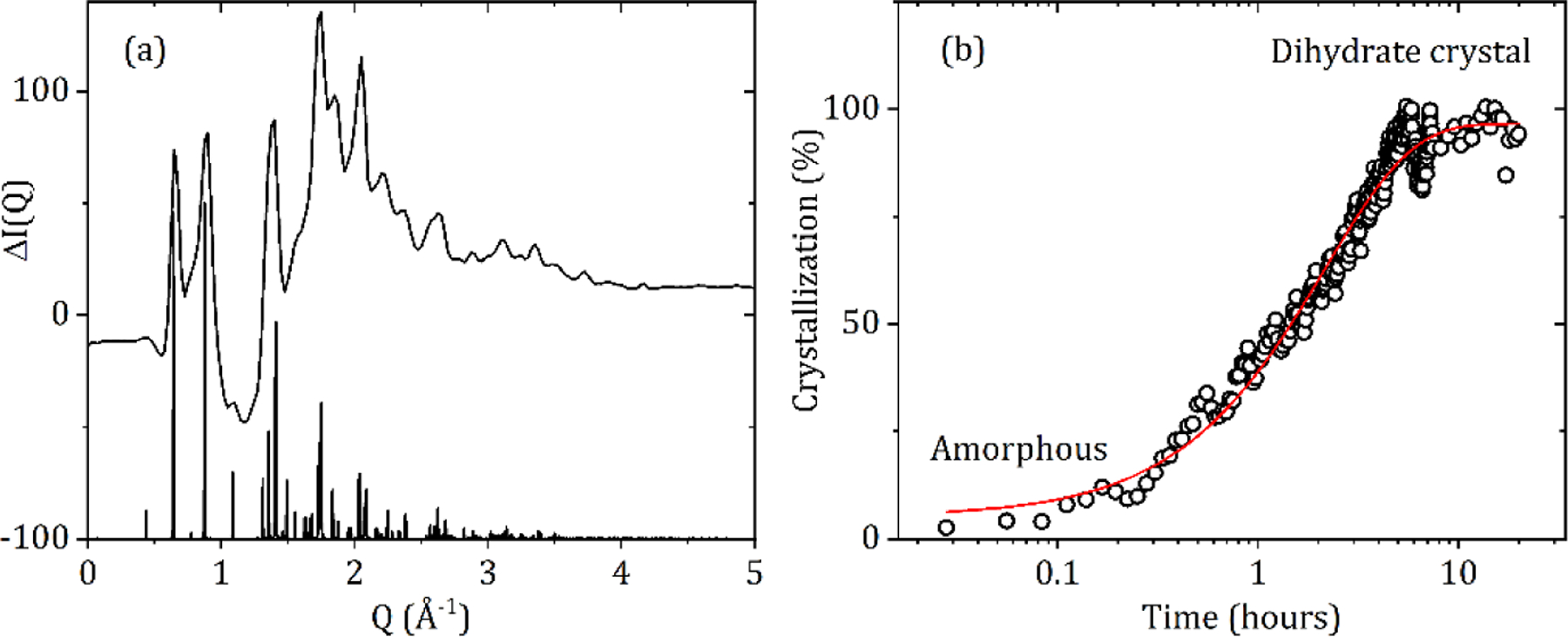
(**a**) The difference in X-ray intensity after 4 h at 80% RH and 21 °C minus the initial amorphous form at 20% RH, shows dihydrate crystal formation upon exposure to high humidity. (**b**) Crystallization rate of amorphous carbamazepine to the dihydrate form at 80% RH and 21 °C fitted with an exponential decay curve with a time constant of 2.16 h.

The Food and Drug Administration reported that carbamazepine can lose up to one third of its effectiveness when stored in humid conditions [26]. Water absorption initiates an amorphous-dihydrate transition and the reverse dihydrate-amorphous transition upon dehydration. The CBZ dihydrate comprises of one carbamazepine molecule and two water molecules, with a network of hydrogen bonds involving the amide group and the water molecules, linking dimers into a double layer [5]. All the hydrophilic parts of the structure are sandwiched within the layer, while the layers contact each other via the hydrophobic parts of the carbamazepine molecules and are connected by van der Waals inter-actions. Our experiments on dry (20% RH) amorphous CBZ suddenly exposed to 80% RH at room temperature directly converted to the dihydrate crystal, see Figure 9. The crystallization rate was determined from the height of the most intense five dihydrate Bragg peaks as a function of time. After an initial exposure of amorphous CBZ for ~30 min the crystallization rate was found to approximately follow an exponential decay curve. The sample saturated after ~4 h, but some variation was still observed in the crystal:glass ratio after that time, most likely due to inhomogeneous packing and swelling of the sample in the region probed by the X-ray beam.

## 5. Conclusions

The EPSR modeling of high energy X-ray diffraction data from liquid and glassy carbamazepine has revealed a mixture of bonding mechanisms, that are also observed in the crystalline forms. However, the variation of these competing interactions between the liquid and glassy forms is shown to be substantial, when analyzed in terms of the associated partial pair distribution functions. Overall, non-bonded aromatic ring (carbon-carbon) interactions at ~2.6 Å are found to be substantially higher in the liquid state. These correlations are likely associated with π-π stacking and face-to-edge contacts between aromatic rings at longer distances. Moreover, the presence of hydrogen bonds per molecule increases by 50% (using an N-O cut off distance of 4.0 Å) upon cooling from the liquid to the glass. Here, we find the lack of crystalline symmetry enables the formation of small groups of isolated hydrogen bonded clusters in the glassy state not observed in the crystalline phases. Consequently, the combination of HEXRD and ssNMR measurements with EPSR modeling represents a powerful combination in the interpretation of liquid and glassy intermolecular structures of small organic pharmaceutical molecules. In addition, in the case of CBZ, the rapidity of the X-ray synchrotron measurements over the timescale of a few minutes is essential, due to the effects of water absorption causing the samples to crystallize in both the liquid and glassy states.

## Supplementary Material

SI

## Figures and Tables

**Table 1.** Parameters used for the Lennard-Jones reference potential in the EPSR simulations. Partial charges were put on the oxygen and methylene hydrogens. No charges were put on the nitrogen atoms.

Atom	Coulomb Charges (*e*)	*ε* (kJ/mole)	*σ* (Å)
H1	+0.25	0.00	0.00
N1 & N2	0.00	0.70	3.20
O1	−0.50	0.65	3.10

**Table 2.** ^13^C chemical shifts of CBZ in CDCl_3_ solution, and ssNMR ^1^H-^13^C CP-MAS chemical shifts of CBZ III, CBZ dihydrate, and CBZ glass. Peak positions were fit using TopSpin 4.0 for CDCl3 solution, CBZ III and dihydrate. CBZ glass peaks were fit using Gaussian deconvolution in OriginPro, see supplementary materials.

NMR Labels for Carbamazepine	Carbon Number	CDCl_3_ Solution	CBZ III	Dihydrate	Glass
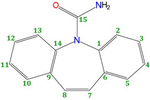	15	157.19	159.0	158.6	159.1 157.6
	1, 14	140.05	140.3 137.2	140.9 140.1	141.9 140.2
	6, 9	135.02	134.7	135.3 134.7	135.0
	7, 8	130.48	133.2	131.2	
	2, 13	129.59	132.1	129.9	
	4, 11	129.47	130.9	129.5	129.3
	5, 10	128.74	129.2	128.9	
	3, 12	127.76	127.3	126.8 126.0	

**Table 3.** Thermodynamic parameters obtained from DSC analysis of CBZ polymorphs and glass.

Structure	T_g_ (°C)	∆H (J/g)	Onset (°C)	T_m_ (°C)
**CBZ I**				

Form I fusion		113.95	190.38	191.38

**CBZ III**				

CBZ III fusion		20.856	173.35	174.95
CBZ I cold-crystallization		5.217	175.44	175.95
CBZ I fusion		110.91	191.10	191.26

**CBZ**·**2H_2_O**				

Dehydration		362.85	73.03	84.49, 95.35
Mixed phase melting		7.085	155.99	164.47
CBZ I fusion		95.052	190.75	191.16

**CBZ glass**				

Glass transition, onset				
Glass transition, midpoint	42.83			
CBZ I cold-crystallization	46.03	57.083	81.98	97.62
CBZ I fusion		102.21	189.00	190.31

## Data Availability

Data is available on request.

## References

[1] Yu L Amorphous pharmaceutical solids: Preparation, characterization and stabilization. Adv. Drug Deliv. Rev 2001, 48, 27–42. 11325475 10.1016/s0169-409x(01)00098-9

[2] Willart JF; Descamps M Solid State Amorphization of Pharmaceuticals. Mol. Pharm 2008, 5, 905–920. 18954076 10.1021/mp800092t

[3] Byrn SR; Zografi G; Chen XS Solid State Properties of Pharmaceutical Material; John Wiley & Sons, Inc.: Hoboken, NJ, USA, 2017.

[4] Healy AM; Worku ZA; Kumar D; Madi AM Pharmaceutical solvates, hydrates and amorphous forms: A special emphasis on cocrystals. Adv. Drug Deliv. Rev 2017, 117, 25–46. 28342786 10.1016/j.addr.2017.03.002

[5] Harris RK; Ghi PY; Puschmann H; Apperley DC; Griesser UJ; Hammond RB; Ma C; Roberts KJ; Pearce GJ; Yates JR; Structural Studies of the Polymorphs of Carbamazepine, Its Dihydrate, and Two Solvates. Org. Process Res. Dev 2005, 9, 902–910.

[6] Billinge S; Dykhne T; Juhas P; Bozin E; Taylor R; Florence A; Shankland K Characterisation of amorphous and nanocrystalline molecular materials by total scattering. CrystEngComm 2010, 12, 1366–1368.

[7] Petkov V; Ren Y; Suchomel M Molecular arrangement in water: Random but not quite. J. Phys. Condens. Matter 2012, 24, 155102. 22418283 10.1088/0953-8984/24/15/155102

[8] Skinner LB; Benmore CJ; Parise JB Comment on ‘Molecular arrangement in water: Random but not quite’. J. Phys. Condens. Matter 2012, 24, 338001; Discussion 338002. 22824868 10.1088/0953-8984/24/33/338001

[9] Soper AK Recent water myths. Pure Appl. Chem 2010, 82, 1855–1867.

[10] Wright AC The Great Crystallite Versus Random Network Controversy: A Personal Perspective. Int. J. Appl. Glass Sci 2014, 5, 31–56.

[11] Benmore CJ; Benmore SR; Edwards AD; Shrader CD; Bhat MH; Cherry BR; Smith P; Gozzo F; Shi C; Smith D; A High Energy X-ray Diffraction Study of Amorphous Indomethacin. J. Pharm. Sci 2022, 111, 818–824. 34890631 10.1016/j.xphs.2021.12.003 PMC11064786

[12] Dołęga A; Juszyńska-Gałązka E; Osiecka-Drewniak N; Natkański P; Kuśtrowski P; Krupa A; Zieliński PM Study on the thermal performance of carbamazepine at different temperatures, pressures and atmosphere conditions. Thermochim. Acta 2021, 703, 178990.

[13] Benmore CJ A Review of High-Energy X-ray Diffraction from Glasses and Liquids. ISRN Mater. Sci 2012, 2012, 852905.

[14] Hammersley AP; Svensson SO; Hanfland M; Fitch AN; Hausermann D Two-dimensional detector software: From real detector to idealised image or two-theta scan. High Press. Res 1996, 14, 235–248.

[15] Qiu X; Thompson JW; Billinge SJL PDFgetX2: A GUI-driven program to obtain pair distribution function from X-ray powder diffraction data. J. Appl. Crystallogr 2004, 37, 678.

[16] Skinner LB; Benmore CJ; Parise JB Area detector corrections for high quality synchrotron X-ray structure factor measurements. Nucl. Instrum. Methods Phys. Res. Sect. A: Accel. Spectrometers Detect. Assoc. Equip 2012, 662, 61–70.

[17] Soper AK Partial structure factors from disordered materials diffraction data: An approach using empirical potential structure refinement. Phys. Rev. B 2005, 72, 104204.

[18] Soper AK Joint structure refinement of X-ray and neutron diffraction data on disordered materials: Application to liquid water. J. Phys. Condens. Matter 2007, 19, 335206. 21694129 10.1088/0953-8984/19/33/335206

[19] Dołęga A; Juszyńska-Gałązka E; Deptuch A; Baran S; Zieliński PM Cold-crystallization and physical stability of glassy carbamazepine. Thermochim. Acta 2022, 707, 179100.

[20] Dołęga A; Zieliński PM; Osiecka-Drewniak N New Insight Into Thermodynamical Stability of Carbamazepine. J. Pharm. Sci 2019, 108, 2654–2660. 30926446 10.1016/j.xphs.2019.03.027

[21] Dołęga A; Zieliński PM Kinetics of non-isothermal cold-crystallization of carbamazepine in the glassy state studied by DSC. J. Non-Cryst. Solids 2022, 575, 121198.

[22] Dołęga A; Krupa A; Zieliński PM Enhanced thermal stability of carbamazepine obtained by fast heating, hydration and recrystallization from organic solvent solutions: A DSC and HPLC study. Thermochim. Acta 2020, 690, 178691.

[23] Naima Z; Siro T; Juan-Manuel G-D; Chantal C; René C; Jerome D Interactions between carbamazepine and polyethylene glycol (PEG) 6000: Characterisations of the physical, solid dispersed and eutectic mixtures. Eur. J. Pharm. Sci 2001, 12, 395–404. 11231106 10.1016/s0928-0987(00)00168-8

[24] Grzesiak AL; Lang M; Kim K; Matzger AJ Comparison of the four anhydrous polymorphs of carbamazepine and the crystal structure of form I. J. Pharm. Sci 2003, 92, 2260–2271. 14603511 10.1002/jps.10455

[25] Suhasini M; Sailatha E; Gunasekaran S; Ramkumaar GR Molecular structure and spectroscopic characterization of Carbamazepine with experimental techniques and DFT quantum chemical calculations. Spectrochim. Acta Part A Mol. Biomol. Spectrosc 2015, 141, 252–262. 10.1016/j.saa.2015.01.059 25682215

[26] Khankari RK; Grant DJW Pharmaceutical hydrates. Thermochim. Acta 1995, 248, 61–79.

[27] Metz G; Ziliox M; Smith SO Towards quantitative CP-MAS NMR. Solid State Nucl. Magn. Reson 1996, 7, 155–160. 9050152 10.1016/s0926-2040(96)01257-x

[28] Johnson RL; Schmidt-Rohr K Quantitative solid-state 13C NMR with signal enhancement by multiple cross polarization. 39 J. Magn. Reson 2014, 239, 44–49. 24374751 10.1016/j.jmr.2013.11.009

