# Supplementary material for: The Structure of Liquid and Glassy Carbamazepine
Source: Quantum Beam Sci. Author manuscript; Available in PMC 2024 May 17. (PMC11099869; doi:10.3390/qubs6040031)
Supplement: SI [file NIHMS1984478-supplement-SI.pdf]

quantum beam science

Supporting Information

The Structure of Liquid and Glassy Carbamazepine

Chris J. Benmore [1,2,*], **Angela Edwards** [2], **Oliver L.G. Alderman** [3], **Brian** R. **Cherry** [2], **Pamela Smith** [4], **Daniel Smith** [4], **Stephen Byrn** [4], **Richard Weber** [5] and **Jeffery L. Yarger** [2]

1 Advanced Photon Source, Argonne National Laboratory, Argonne, IL 60439, USA
2 School of Molecular Sciences, Arizona State University, Tempe, AZ 85281, USA
3 Rutherford Appleton Laboratory, Harwell Campus, Didcot, OX11 0QX, UK
4 Improved Pharma, West Lafayette, IN 47906, USA
5 Materials Development, Inc., Arlington Heights, IL 60004, USA
* Correspondence: benmore@anl.gov

A simple gaussian deconvolution was performed in OriginPro using the peak analyzer tool. Peak center parameters were constrained using the chemical shift values from Harris et al.[1] with a convergence to $R^2 = 0.997$ after 19 iterations (Fig. S1). Attempts to deconvolute the peaks at 135.0 and 129.3 ppm did not converge within 6000 iterations and would yield a peak with zero or near zero area, so these peaks were assigned only one peak position, though they account for multiple carbons.

The template details the sections that can be used in a manuscript. Note that each section has a corresponding style, which can be found in the "Styles" menu of Word. Sections that are not mandatory are listed as such. The section titles given are for articles. Review papers and other article types have a more flexible structure.

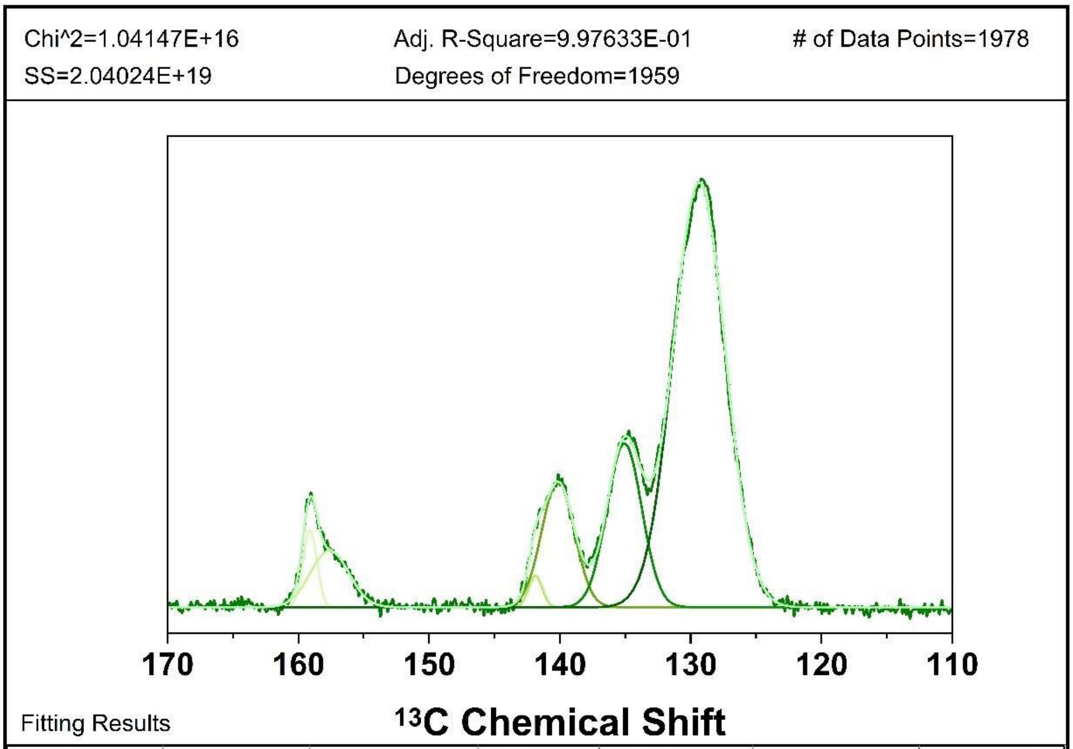

Fitting Results

Peak Index	Peak Type	Area Intg	FWHM	Max Height	Center Grvty	Area IntgP
1	Gaussian	2.16415E9	1.25837	1.61565E9	159.11039	3.00283
2	Gaussian	4.40617E9	3.47322	1.19178E9	157.60969	6.11372
3	Gaussian	8.66463E8	1.22569	6.64108E8	141.86889	1.20225
4	Gaussian	8.34535E9	2.96278	2.64614E9	140.13956	11.57946
5	Gaussian	1.15658E10	3.14422	3.45567E9	135.06904	16.04799
6	Gaussian	4.47223E10	4.68567	8.96644E9	129.34912	62.05376

Figure S1. Gaussian deconvolution of ^{1}H-^{13}C CP-MAS spectrum of amorphous CBZ to identify shoulder peak positions.

While it is well known that ^{1}H-^{13}C CP-MAS is only quantitative when using specific conditions, due to factors such as the efficiency of magnetization transfer rates with different heteronuclear dipolar interaction strengths within the sample, and the effects of sample spinning on molecular motion (especially relevant for a glass with frictional heating within 20ºC of T_g) which average dipolar couplings.[2,3] Cross polarization times constants (T_{CH})are generally longer for more mobile carbons, so the differences among the carbons 1-14 in CBZ are expected to be low. Carbon 15, the carboxamide carbon (see table 2 of the main text) which is of primary interest due to the functional groups which participate in the dimer/oligomer formation via hydrogen bonding in the liquid and glass states, is the most mobile and thus expected to have the longest T_{CH}. For this reason, the contact time was chosen to be relatively long, at 1.5 ms, maximizing the ratio of the carboxamide peak to the other peaks. Thus, this peak area should not be considered quantitative out of the sum of peak areas, but we can consider the ratio between the peak areas for the two carboxamide peaks, finding that the downfield peak at 159.1 ppm which fits within the dimer ^{13}C chemical shift ranges to the broader shoulder at 157.6 ppm which is between the solution and dihydrate ^{13}C chemical shifts to be approximately 1:2. The results from high energy x-ray diffraction experiments are ~38% dimer, with the remaining percentages a sum of 0 and 2-4 neighbor monomers and oligomers, respectively. Given the lower mobility expected for states which are extensively hydrogen bonded, the contribution of monomers that have highest mobility most likely accounts for the slightly higher than expected area of the upfield shoulder peak. Thus, the ssNMR data, taken together with the other characterizations, support the high-energy x-ray diffraction measurements and ESPR modeling.

References

1. Harris, R.K.; Ghi, P.Y.; Puschmann, H.; Apperley, D.C.; Griesser, U.J.; Hammond, R.B.; Ma, C.; Roberts, K.J.; Pearce, G.J.; Yates, J.R.; et al. Structural Studies of the Polymorphs of Carbamazepine, Its Dihydrate, and Two Solvates. *Org. Process Res. Dev.* **2005**, *9*, 902–910.
2. Metz, G.; Ziliox, M.; Smith, S.O. Towards quantitative CP-MAS NMR. *Solid State Nucl. Magn. Reson.* **1996**, *7*, 155–160.
3. Johnson, R.L.; Schmidt-Rohr, K. Quantitative solid-state 13C NMR with signal enhancement by multiple cross polarization. *J. Magn. Reson.* **2014**, *239*, 44–49.
